# Beyond paralysis: Impact of spinal cord injury on brain inflammation and cognitive function through cell therapy

**DOI:** 10.4103/NRR.NRR-D-25-00520

**Published:** 2025-09-03

**Authors:** Quentin Delarue, Nicolas Guérout

**Affiliations:** Université Paris Cité, CNRS UMR, Saints-Pères Paris Institute for Neurosciences, Paris, France

Traumatic spinal cord injury (SCI) is a pathological condition that impairs both sensorimotor and cognitive functions. While research has long focused on understanding the pathophysiology of SCI and developing treatments, only a few studies have investigated the cellular and molecular consequences that occur in the brain after trauma. From the earliest stages, the injury triggers microglial activation, increased neuronal death, and reduced hippocampal neurogenesis in the dentate gyrus, which in turn leads to cognitive impairments such as deficits in working memory, attention, learning capacity, and the detection and evaluation of stimuli (Li et al., 2020). These effects are a direct consequence of secondary damage following SCI and the onset of neuroinflammation, which can persist chronically.

Based on these descriptions, we aimed to investigate the potential effects of cell transplantation on the hippocampus of mice with SCIs, given the cells’ proven effectiveness in promoting tissue regeneration and functional recovery (Chen et al., 2024). We transplanted olfactory ensheathing cells (OECs) into mice following SCI and subsequently evaluated their exploratory behavior using the novel object recognition test. This test involves a confined environment divided into four equal zones, two zones containing objects and two empty zones. In the first phase, both objects are identical; in the second phase, one object is replaced with a novel item that differs in size, shape, texture, and color. The mice’s ability to explore the object zones and distinguish the novel object was then assessed. Our results revealed that 64 days post-injury, the transplanted mice displayed a significantly enhanced capacity to explore their environment, spending up to twice as much time in the object zones compared to non-transplanted mice. Notably, the total distance traveled was identical between the two groups, indicating that the improved exploratory behavior was due to an enhanced ability to detect and evaluate their surroundings, behavior comparable to human curiosity (Delarue et al., 2025).

To determine the cellular basis of the observed cognitive improvements, we conducted immunohistochemical analyses of the hippocampus in these mice. Our findings revealed increased neurogenesis in the dentate gyrus, as evidenced by a higher density of immature doublecortin positive (DCX^+^) neurons 14 days post-injury and a greater density of neuron-specific nuclear protein positive (NeuN^+^) mature neurons 64 days post-injury in the transplanted group. Although we did not specifically analyze the maturation of the DCX^+^ neurons, it is plausible that the increased density of mature neurons is a consequence of enhanced neurogenesis. Additionally, the elevated neuronal density at 64 days in transplanted animals may also be attributed to improved modulation of microglial reactivity, as indicated by a reduction in the density of Iba1^+^/CD86^+^ cells (Delarue et al., 2025). Indeed, microglial activation creates a pro-inflammatory environment that is detrimental to neuronal survival. Specifically, when microglia are activated in a pro-inflammatory manner, they produce tumor necrosis factor alpha and interleukin-1β (IL-1β), which can trigger neuronal apoptosis (Muzio et al., 2021).

We also examined astrocyte density. Although no significant difference was observed, the trend toward increased density in transplanted animals suggests that the control of neuroinflammation associated with spinal cord transplantation may help preserve the physiological functions of these cells in supporting neurons. Indeed, astrocytes contribute to neurogenesis and neuronal survival by secreting neurotrophic factors, particularly glial-derived neurotrophic factor and brain-derived neurotrophic factor, and play a key role in maintaining synaptic function by regulating neurotransmitter levels, such as glutamate, which can lead to excitotoxicity, as well as maintaining ionic homeostasis essential for proper action potential propagation. Moreover, astrocytes are crucial in modulating synaptic plasticity by strengthening neuronal connections, thereby facilitating long-term potentiation and learning (Allen and Eroglu, 2017).

In our study, it is challenging to determine precisely how OEC transplantation improves cerebral neuroinflammation and enhances cognitive abilities in mice. It is possible that these effects result from improved tissue regeneration at the lesion site. Following transplantation, OECs promote tissue repair and reduce spinal inflammation, thereby limiting secondary damage caused by cytokines such as tumor necrosis factor alpha and IL-1β produced by microglial cells and astrocytes. After SCI, these cytokines can spread via the CSF, glymphatic system, and bloodstream, ultimately triggering cerebral neuroinflammation. Specifically, by binding to receptors on endothelial cells of the blood–brain barrier, these cytokines increase blood–brain barrier permeability, facilitating their entry into the parenchyma, where they directly affect cerebral microglial cells, and also stimulate the recruitment and infiltration of immune cells into the parenchyma (Sweeney et al., 2018).

Furthermore, transplanted OECs secrete anti-inflammatory cytokines such as IL-4 and IL-10 (Chen et al., 2024). To our knowledge, no study has yet established a direct correlation between the production of these molecules by OECs and their presence in the CSF or bloodstream. However, these molecules can reach the cerebral parenchyma either by binding to receptors on the endothelial cells of the blood–brain barrier or through passive diffusion via the glymphatic system (Muzio et al., 2021), thereby reducing neuroinflammation and potentially promoting neuronal survival and neurogenesis. Consequently, the secretion of these beneficial molecules within the spinal cord may contribute to creating a more favorable cerebral environment by mitigating neuroinflammation and fostering conditions that support neuronal survival.

Since OECs are CNS cells, we wondered whether transplanting cells derived from peripheral tissue would yield the same therapeutic effects as OECs. Therefore, we transplanted mesenchymal stem cells after SCI and observed similar beneficial outcomes. Mesenchymal stem cell transplantation reduced microglial reactivity, thereby decreasing neuroinflammation and promoting an increase in hippocampal neuronal density, particularly in the dentate gyrus. Behaviorally, these tissue-level improvements correlated with a reversal of the injury-induced cognitive deficits; during the novel object recognition test, the transplanted mice demonstrated an enhanced ability to explore their environment and interact with objects (Delarue et al., 2025).

Neurogenesis impairment following SCI is not confined to the hippocampus; it has also been observed in the olfactory bulbs (OBs). Felix et al. (2012) demonstrated that the migration of neuroblasts from the subventricular zone via the RMS and their differentiation into olfactory neurons are reduced. They also reported that there is no change in astrocyte or microglial cell reactivity 90 days after SCI (Felix et al., 2012). However, no study has yet examined astrocytosis or microgliosis in the OBs during the days or weeks following SCI. Nonetheless, similar to the hippocampus, it is highly likely that microglial cells in the OBs contribute to the inflammatory response. Consequently, cell transplantation could potentially reduce neuroinflammation in the OBs as well.

These findings underscore the importance of approaching SCI treatment holistically, taking into account the limitation of secondary damage that can have significant cerebral and peripheral consequences. Indeed, numerous peripheral effects associated with SCI have been described as a result of systemic immune activation. The dissemination of tumor necrosis factor alpha, IL-1β, and IL-6 into the bloodstream can lead to disturbances in organs such as the spleen, kidneys, lungs, and liver (Anthony and Couch, 2014), exacerbating the overall dysregulation of homeostasis can lead to secondary pathologies and negatively affect spinal cord regeneration. Furthermore, as we have observed, all tissues exhibit an inflammatory state and cellular dysfunction. In human cell transplants, which are performed autologously, the quality of the transplanted cells and the potential to enhance their therapeutic capacity become critical considerations. Indeed, it is possible that these cells are dysfunctional, which could prevent them from providing the maximal therapeutic effect in both the spinal cord and the brain.

Therefore, it would be interesting to continue this research alongside studies aimed at developing a comprehensive therapeutic solution for SCI, one that addresses all the consequences of the pathology, to ultimately create an effective treatment. By targeting both peripheral and cerebral disturbances, we can enhance quality of life of patients and limit the emergence of secondary pathologies. Moreover, any effective therapy must be paired with functional rehabilitation, as the intensity of such rehabilitation is partly correlated with motivation, which in turn is linked to anxiety and depression. Thus, improving the cognitive state of patients is as crucial as intervening directly on the spinal tissue.

It is now imperative that future clinical research projects, particularly those involving cell therapy, assess cognitive functions of patients both before and after cell transplantation to determine whether the findings observed in mice are also present in humans with SCI. Beyond advancing scientific understanding, this approach would provide an objective measure of the success of cell transplantation or other treatments using cognitive parameters.

**Figure 1:**
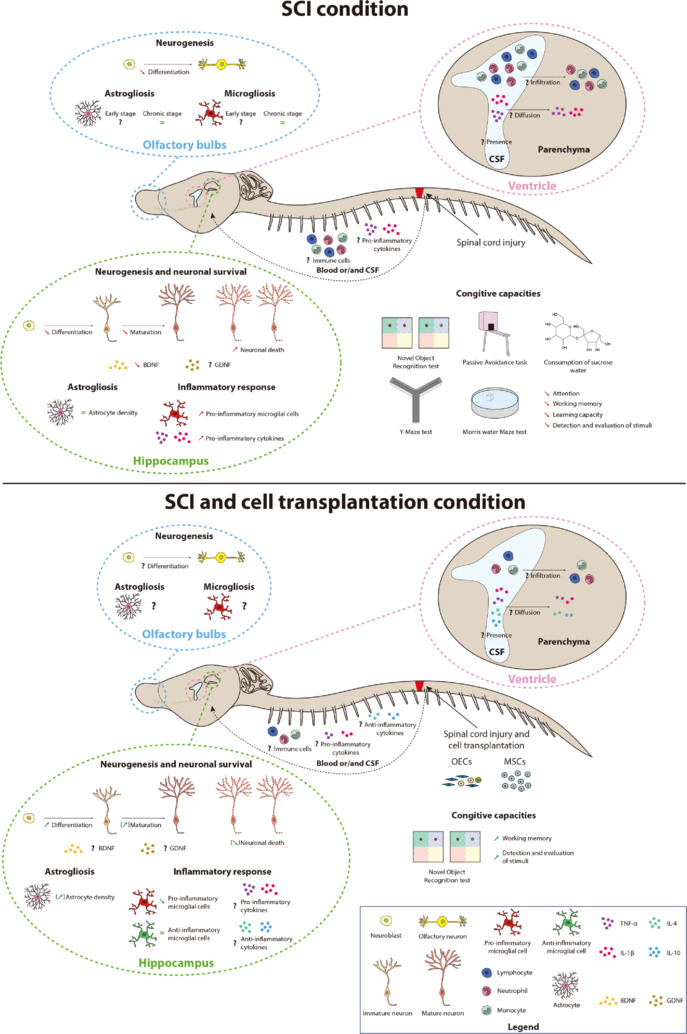
Summary of the main results of the study of Delarue et al. (2025). BDNF: Brain-derived neurotrophic factor; CSF: cerebrospinal fluid; GDNF: glial cell line-derived neurotrophic factor; IL-1β: interleukin-1 beta; IL-4: interleukin-4; IL-10: interleukin-10; MSCs: mesenchymal stem cells; OECs: olfactory ensheathing cells; TNF-α: tumor necrosis factor alpha.

The paper by Delarue et al. (2025) also highlights the important yet underexplored link between neuroinflammation, cognition, and SCI (**Figure 1**). To our knowledge, Wu et al. (2014) were the first to characterize the connection between SCI and cognitive deficits. Since then, several studies have reinforced this interaction, demonstrating that cognitive decline following SCI is influenced by both sex and age, and that it increases the risk of developing dementia later in life (Lei et al., 2024). However, the reverse relationship has not yet been investigated, highlighting the fundamental and reciprocal connections between the spinal cord and the brain. This point is particularly significant as it reintroduces a rarely used term in neuroscience: “neuraxis,” an outdated yet relevant concept that views the spinal cord and brain as a single anatomical and functional unit rather than as two separate, independent organs.
